# Multisectoral nutrition planning in Nepal: Evidence from an organizational network analysis

**DOI:** 10.1111/mcn.13112

**Published:** 2021-03-04

**Authors:** Jenny Ruducha, Amiya Bhatia, Carlyn Mann, Harriet Torlesse

**Affiliations:** ^1^ Braintree Global Health Vancouver British Columbia Canada; ^2^ Department of Global Health and Development London School of Hygiene and Tropical Medicine London UK; ^3^ Nutrition Section UNICEF Regional Office for South Asia Kathmandu Nepal

**Keywords:** case study, intersectoral collaboration, organizational network analysis, qualitative research, social networks, Nepal, undernutrition

## Abstract

Multisectoral approaches are central to the global Scaling Up Nutrition (SUN) movement and the Sustainable Development Goals. Nepal joined SUN in 2011 and approved the first 5‐year Multisectoral Nutrition Plan (MSNP) in 2012, covering 2013–2017. This mixed methods study draws on organizational network analysis (ONA) and qualitative interviews with a sample of 22 organizations to examine (1) levels of engagement and network dynamics among government sectors and development organizations and (2) milestones and processes in the development and implementation of Nepal's MSNP. Findings suggest that the development of the MSNP was related to the high density of organizational connections; the leadership role of the Nepal's National Planning Commission and the National Nutrition and Food Security Secretariat; and the bridging roles played by a few government ministries and UN agencies that linked organizations that did not have direct relationships with each other. Specialized roles were observed for the three types of working relationships: policy dialogue, strategic planning and implementation. Partners were less connected on MSNP implementation than for policy dialogue and strategic planning, which may have constrained collaborative scale‐up efforts. The Ministry of Agricultural Development, in particular, was the conduit for connecting non‐health sectors into the broader network. Our study offers insights into the structure and dynamics of multisectoral planning in Nepal. It also contributes to a small but growing literature that illustrates how ONA can be applied to measure and use network results to elucidate the processes for strengthening multisectoral planning and implementation of nutrition‐specific and nutrition‐sensitive interventions.

**Key messages**

- The development of Nepal's Multisectoral Nutrition Plan led to the establishment of strong governance structures at the national level and fostered a high level of organizational connectivity between sectors and stakeholders.
- The agriculture sector can play a larger bridging role in mobilizing non‐health sectors to participate in broader multisectoral networks for nutrition.
- Analysing organizational network structures and relationship dynamics using organizational network analysis (ONA) can open up the ‘black box’ of network dynamics to strengthen multisectoral collective action.

## 1 INTRODUCTION

Multisectoral approaches for nutrition feature prominently in the strategies and plans of governments in middle‐ and low‐income countries. Although such approaches were adopted as early as the 1970s, they often achieved short‐lived results due to wavering political support, a lack of sustainable resources and inadequate institutional capacity (Levinson & Balarajan, 2013; Warren & Frongillo, 2017). However, multisectoral approaches were revived in the last decade in response to growing evidence of the impacts of malnutrition on child survival, development and economic growth (Black et al., 2013), the need for progress on nutrition to achieve multiple Millennium Development Goals (MDGs) (UNICEF, 2009) and Sustainable Development Goals (SDGs) (Development Initiatives, 2017) and the recognition that a combination of nutrition‐specific and nutrition‐sensitive interventions is needed to tackle the multiple determinants of malnutrition (Bhutta et al., 2013; Black et al., 2013; Ruel, Quisumbing, & Balagamwala, 2018).

The global Scaling Up Nutrition (SUN) movement in 2010 brought greater attention to nutrition governance in achieving a sustainable reduction in malnutrition (Acosta & Fanzo, 2012). It mobilized multiple stakeholders—governments, donors, UN agencies, civil society and the private sector—in collective multisectoral country‐led efforts to improve nutrition. Countries that join the SUN movement are encouraged to establish multisectoral and multi‐stakeholder platforms to coordinate across all relevant sectors and constituencies and to align actions around costed country plans (Scaling up Nutrition Movement, 2012). Of the 61 countries that joined the SUN movement, 55 have a multi‐stakeholder coordination platform, and 42 have a multi‐year nutrition plan (Scaling up Nutrition Movement, 2019).

Although there is a strong case for acting across several sectors to improve nutrition, documented experience on how to design, plan and scale up multisectoral approaches is quite rare (The World Bank, 2013; World Health Organization, 2013), although some recent case studies have begun to explore this area (Cunningham, Headey, Singh, Karmacharya, & Rana, 2017; Garrett & Natalicchio, 2011; Levinson et al., 2013; Pelletier, Gervais, Hafeez‐ur‐Rehman, Sanou, & Tumwine, 2016). The shortage of empirical research on multisectoral collaboration is juxtaposed with a high level of global interest that underscores the importance and timeliness of advancing this knowledge base (Glandon et al., 2019).

Nepal is an active participant in many global initiatives and has a long history of implementing multisectoral nutrition plans (Pomeroy‐Stevens et al., 2016). This began with the development of nutrition strategies (1978 and 1986), the National Plan of Action for Nutrition (NPAN) in 1998 and an integrated plan of action in 2006 (Shrimpton, Crum, Basnet, Mebrahtu, & Dahal, 2014). Building on these efforts and a commitment to meeting the MDG target to half the prevalence of child underweight by 2015, Nepal joined the SUN movement in 2011 and launched the first 5‐year Multisectoral Nutrition Plan (MSNP) in 2012 with a 5‐year plan (2013–2017) as part of a 10‐year vision to reduce stunting. The goal of the MSNP was to ‘improve human capital, especially among the poor segments of society, to improve maternal and child nutrition and health’, with a purpose to ‘strengthen the multi‐sector efforts of the NPC and other stakeholders to foment capacity development for improved nutrition at all levels of society in Nepal’ (Government of Nepal National Planning Commission, 2012). The first MSNP was followed by a second 5‐year MSNP, covering 2018–2022.

As Nepal's leadership in multisectoral nutrition planning is gaining global recognition, it becomes more critical to systematically identify the drivers of success and weaknesses of the MSNP's development and implementation. We designed a mixed methods study to examine the structure and working relationships of the organizations that contributed to the development first MSNP with a focus on the role of partnerships in strengthening the multisectoral platform to improve nutrition. The main objectives were (1) to determine the levels of engagement and network dynamics among key organizations and sectors of government and (2) to understand critical milestones and processes in the development of Nepal's MSNP. Our study contributes to a small but growing literature illustrating the way organizational network analysis (ONA) can be adopted to measure and use network results as an input into the ‘black box’ of the processes and dynamics to promote multisectoral strengthening for more effective implementation.

## 2 METHODS

### 2.1 Study design

Our primary method was ONA, supported by open‐ended questions to understand the catalytic events, milestones and organizational partnerships leading to the development of the MSNP. Reports and grey literature were also reviewed to supplement the data on the key events and triggers that enabled multisectoral collaboration.

ONA is a social network method that is grounded in a sociological paradigm based on the premise that political, social and economic actors can be perceived as social networks of relations made up of interrelated units (i.e. actors or organizations) (Faust & Wasserman, 1994; Scott & Carrington, 2011). ONA addresses the limitations of standard frameworks, tools and methods in studying the complexity of multisectoral collaboration (Bennett, Glandon, & Rasanathan, 2018; Glandon, Meghani, Jessani, Qiu, & Bennett, 2018) and offers a novel relational approach to identify the network structure and the roles of key organizational actors in multisectoral collaborations. The results go beyond organizational charts to detect underlying dynamics that may not always correspond to reported structures and official roles.

### 2.2 Identification of sample

A review of organizations engaged in the development and implementation of the MSNP and consultations with the UNICEF regional and Nepal offices was used to create a bounded list of 22 organizations in line with ONA processes (Dozier et al., 2014). For each selected organization, up to three key informants who had been involved in one or more stages in the development of the MSNP were identified and listed in order of priority for data collection. Most of the interviews were conducted with one organizational representative; however, other individuals from the same organization were also interviewed if the primary respondent did not feel equipped to answer some questions.

Respondents included eight government organizations; eight UN agencies or programme sections; two donors; and four organizations representing civil society and national and international NGOs (Table 1—further information on organizations and the nutrition architecture in Nepal can be found in Shrimpton et al., 2014). A total of 17 respondents also answered qualitative interview questions. Five organizations did not participate because respondents lacked time or had recently joined their post and were not able to answer questions about the MSNP.

**Table 1:** Organizations interviewed for the MSNP ONA and qualitative survey^a^

Type	Organization	Acronym	ONA	Qualitative
Civil society/NGO/INGO *n* = 4	Civil Society Alliance for Nutrition	CSA_Nut	•	•
	Helen Keller International	HKI	•	•
	Save the Children	Save	•	
	Suaahara Project^b^	Suaahara	•	•
Donor *n* = 2	European Union	EU	•	
	US Agency for International Development	USAID	•	•
Government *n* = 8	Ministry of Agriculture Development	MoAD	•	•
	Ministry of Education	MoE	•	
	Ministry of Federal Affairs and Local Development	MoFALD	•	•
	Ministry of Health and Population, Department of Health Services	MoHP, DHS	•	•
	Ministry of Health and Population, Nutrition Section, Child Health Division^c^	MoHP_NutCH	•	•
	Ministry of Urban Development	MoUD	•	
	National Nutrition and Food Security Secretariat^d^	NNFSS	•	•
	National Planning Commission	NPC	•	•
UN *n* = 8	Food and Agriculture Organization	FAO	•	•
	UNICEF, Child Friendly Local Governance	UNICEF_CFLG	•	•
	UNICEF, Education	UNICEF_Edu	•	•
	UNICEF, Nutrition	UNICEF_Nut	•	•
	UNICEF, Social Policy and Economic Analysis	UNICEF_SPEA	•	•
	World Bank	WB	•	
	World Food Programme	WFP	•	•
	World Health Organization	WHO	•	•
Total			22	17

^a^ Information on organizations and the nutrition architecture in Nepal can be found in Shrimpton et al. (2014).

^b^ The Suaahara I Project was identified as a distinct entity involved in the MSNP. It was funded by USAID and implemented by Save the Children in coordination with many implementing partners including Helen Keller International.

^c^ The Ministry of Health and Population, Nutrition Section, Child Health Division is located in the Ministry of Health and Population, Department of Health Services.

^d^ The National Nutrition and Food Security Secretariat is located in the National Planning Commission.

### 2.3 Data collection

A structured ONA questionnaire was administered to capture basic information about individual and organizational roles and network dynamics including strength and intensity of relationships between organizations involved in developing the MSNP. The first ONA question established whether there was a relationship between two organizations and, if so, whether there was any working relationship related to the MSNP. If a relationship was reported, further questions explored whether this working relationship involved (1) policy dialogue and development, (2) strategic planning and (3) implementing the scale‐up of the plan. The three processes involved in joint MSNP development were explored with organizations in a pretest stage of the instrument development. The three final components followed a logical continuum from (1) discussing ideas about developing an MSNP; (2) setting goals and priorities through a strategic planning process; and (3) developing an implementation plan and scale‐up.

Following the completion of the ONA questions, respondents were asked a set of open‐ended questions using a semi‐structured interview guide. Respondents were asked to describe catalytic events, the major milestones of progress, the timing of their occurrence, who was involved and how these contributed to the development or implementation of the MSNP. Finally, respondents were asked how the global, country and local environment, finances and human resources helped or hindered MSNP development and to reflect on lessons learned.

All interviews were conducted by an external investigator engaged by the UNICEF Regional Office for South Asia in March 2015. In a few cases, a Nepali translator was also employed. The duration of each interview ranged from 45 to 90 min, and most were recorded after obtaining an informed consent.

### 2.4 Data analysis

ONA data were entered in Excel files and constructed into matrices for each ONA measure. UCINET software Version 6 (Borgatti, Everett, & Freeman, 2002) was used to analyse the data, and NetDraw (Borgatti, 2002) enabled creation of visual plots. If key informants from both organizations acknowledged the relationship, this formed a confirmed relationship and improved the reliability of the data. For example, the key informant from Organization A must indicate a relationship exists with Organization B, and key informant from Organization B must indicate a relationship with Organization A for a confirmed relationship to be recorded.

The intensity of relationships was measured by asking key informants whether the nature of their relationship amounted to communication (lowest level of intensity), coordination, collaboration or integration (highest level of intensity). These measures are derived from a review of organizational partnership frameworks that define a continuum of integration from low to high (Gajda, 2004; Ruducha & Jadhav, 2018). A minimum confirmation process was used to verify the relationship intensity. For example, if Organization A indicated that they coordinated with Organization B on the development of the MSNP, whereas Organization B indicated they collaborated with Organization A, then the confirmed intensity of the relationship was set as coordination. See Table 2 for a complete list and definitions of all ONA measures.

**Table 2:** Definitions of network measures (Freeman, 1978; Hanneman & Riddle, 2005)

Node or individual organizational ties
*Degree centrality* is calculated by counting the number of adjacent links to or from an organization. Based solely on direct connections, it reflects the potential power of having direct relationships. These direct links reduce the reliance on intermediaries to access information or resources. The assumption is that more connections are better than fewer connections.
*Betweenness centrality* measures the extent to which organizations fall between pairs of other organizations or individuals on the shortest paths (geodesics) connecting them. This measure represents potential mediation or flow of information or resources between organizations in the network. It is used to assess the power in networks, as an organization may control the flow of information and potential resources, thereby increasing dependence of others who are not directly connected in the network.

Relationship‐level ties
*Multiplexity* is a measure that describes multiple relationships among the same set of organizations. The measures of the relations can be directed or not, and the relations can be recorded as binary, multi‐valued nominal or valued (ordinal or interval). In this study, three types of relationships were specified: advocacy and policy, strategic planning and scale‐up. So the multiplexity score was either 1 (if only one type of linkage existed), 2 (any combination of two linkages) or 3 (all three relationships were confirmed).
*Intensity* describes the level of interaction between different organizations or nodes. The levels of interactions were classified as communication, coordination, collaboration or integration, in order of increasing intensity: *communication* (interaction as necessary to inform others or to check on specific issues); *coordination* (moderate‐intensity interaction to share new ideas, ensure that duplication/overlap is minimized, etc.); *collaboration* (a close, ongoing, reciprocal, working relationship); and *integration* (organizations are part of a unified entity as they share decision‐making, financial and/or human resources and have common policies and procedures).

Network‐level ties
*Centralization* is an expression of how tightly the network structure is organized around its most central point. The general procedure involved in any measure of graph centralization is to look at the differences between the centrality scores of the most central point and those of all other points. Centralization, then, is the ratio of the actual sum of differences to the maximum possible sum of differences.
*Density* is defined as the sum of the ties divided by the number of possible ties (i.e. the ratio of all tie strength present to the number of possible ties). The density of a network may give us insights into phenomena such as the speed at which information diffuses among the nodes and the extent to which actors have high levels of social capital and/or social constraint.

The colours of the nodes in the plots represent the types of organizations in the network, and the size of the nodes was adjusted for betweenness centrality. The acronyms and organizational categories listed in Table 1 were used in constructing the visual plots.

**Figure 1:**
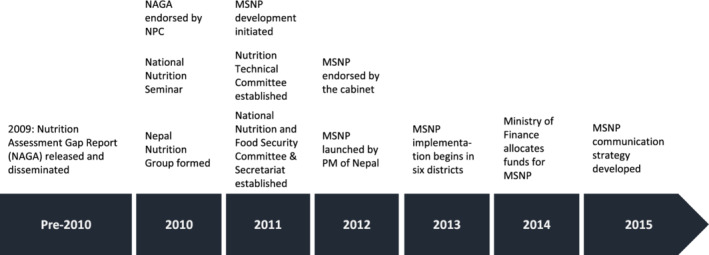
Key milestones in the development and implementation of the Multisectoral Nutrition Plan (MSNP) in Nepal, 2009–2016. Sources: Nepal MSNP, NAGA report, interviews with key informants

Qualitative data were abstracted into a predesigned matrix that was organized by key themes: advocacy and policies; government and partners; initiation of policy change; process timeline for key events; achievements and successes; and lessons learned. Additional themes were also documented and included information about the monitoring and evaluation of MSNP, key challenges and governance processes. The information from interviews as well as the literature/document review was used to construct a timeline for the development of the MSNP (Figure 1) to document the sequence of events that led to the development of the MSNP.

All information from the qualitative interviews and ONA instruments was password protected, and personal identifiers were not used. Data were cleaned, and any potential inconsistencies were double‐checked with paper‐based instruments and audio recordings.

### 2.5 Ethical considerations

UNICEF and BGH reviewed the study, and it met with standards of research for their respective institutions that were set by US mandate for IRBs. Our study posed minimal risk to participants; they were not a vulnerable group; and we obtained informed consent that included information about the study purpose, processes, contact information (if questions arose) and how the data would be used and protected to maintain respondent confidentiality.

## 3 RESULTS

### 3.1 Catalytic events: Organizational actors and milestones

Figure 1 shows the key milestones in the development and implementation of the MSNP. Several respondents underscored how, prior to the development of MSNP, nutrition programming in Nepal was driven by the health sector, with little engagement from other sectors. As one respondent explained, ‘there was an isolated nutrition agenda … people thought it [nutrition] belonged to the health sector and they needed convincing’ (NGO). Another respondent described how previous efforts to develop a multisectoral approach did not gain momentum:

The idea of a multisectoral approach was implemented in the 1980s and was discontinued. In 2009–2010, there was an exercise of a Nutrition Assessment and Gap Analysis and a group of experts developed a report which resulted in the MSNP and pointed out the need for a multisectoral approach to improve nutrition. (NGO)

Respondents almost unanimously identified the contribution of the 2009 Nutrition Assessment and Gap Analysis (NAGA) (Nutrition Assessment Team, 2009) as the foundation for multisectoral planning, programming and financing in Nepal. In describing the antecedents to NAGA, respondents underscored how the results of the 2006 Demographic Health Survey, which indicated that nearly half of Nepalese children were stunted, initiated conversations between the Ministry of Health (MoH), UNICEF and other development partners.

The NAGA was conducted by the Child Health Division, Ministry of Health and Population (MOHP), along with UNICEF, WHO, USAID and the World Bank to synthesize evidence that could be used to develop the MSNP. These organizations conducted sector reviews in 2011 and 2012 to identify the roles of different sectors (e.g. health, education, WASH and agriculture) in addressing nutrition through what was described by several respondents as a collaborative process: ‘I've never worked on an issue where all the donors spoke the same language’ (Donor).

Findings of the NAGA highlighted that nutrition was a multisectoral issue and emphasized that addressing stunting was beyond the capacity of a single ministry. Moreover, NAGA findings suggested that to address the determinants of stunting holistically, there was need to integrate both nutrition‐specific and nutrition‐sensitive interventions into existing development programmes. Respondents further described how the increasing momentum for multisectoral approaches in Nepal occurred alongside global efforts, including the call to address child stunting by the SUN movement, which further reinforced the importance of developing the MSNP.

Following the launch of the NAGA report, the National Nutrition Seminar was held in October 2010 and affirmed the need for a multisectoral nutrition plan (Government of Nepal National Planning Commission, 2012). Other groups were also convened, including the Nepal Nutrition Group in 2010 and a Nutrition Technical Committee in 2011, which was led by the Child Health Division of the MoHP and included experts from government ministries, development partners and academic institutions. The overall aim of the Nutrition Technical Committee was to ‘provide advisory support on nutrition to key sectors, and to monitor performance with respect to nutrition against the goals, objectives and targets in sector strategies and policies’ (Government of Nepal Nutrition Section Child Health Division, 2011).

Respondents perceived the government to be open to new ideas and willing to take on the challenges of a multisectoral approach. In 2011, the development of the MSNP was initiated by the National Planning Commission (NPC), which brought together key government ministries—Agriculture Development, Education, Health and Population, Federal Affairs and Local Government, Finance, Urban Development and Women, Children and Social Affairs—and development partners. The architects of the MSNP drew on global evidence on nutrition interventions, the NAGA report and sector‐specific reviews prepared by the technical reference groups for each sector. As one respondent described:

If we put [in] effort, we can bring all the actors together … each sector has realized that their sectoral contribution is essential to address malnutrition—this realization is the most important. (UN)

The MSNP was approved by the Council of Ministers in June 2012 and launched nationally in September 2012. Several committees were established to lead coordination and implementation at the national and subnational levels. The High Level Nutrition and Food Security Steering Committee (Government of Nepal National Planning Commission, 2012) chaired by the Vice Chair of the NPC was established in 2011 and supported by UNICEF, WFP and the World Bank. Concurrently, a Secretariat was formed and led by the joint UN initiative, Renewed Efforts Against Child Hunger and Undernutrition (REACH). The Steering Committee and Secretariat served as the nodal body to implement MSNP and provide technical assistance to each government ministry to incorporate nutrition programming. Technical assistance included capacity building, information management, communication and advocacy. Government ownership and leadership combined with donor support were considered important to the development and implementation of MSNP.

The leadership role of the government, particularly under the Child Health Division [of the Ministry of Health and Population] and the NPC [National Planning Commission] … they had full ownership of this since day one … if that wasn't there, even if the donors spoke the same language, it [MSNP] would not have happened. (Donor)

Respondents also discussed how the key ministries coordinated to ensure effective programme implementation of an integrated set of essential nutrition interventions (both nutrition specific and nutrition sensitive), which were monitored through a joint management information system. However, a few respondents also described some tensions between sectors working together as organizational priorities were not always aligned as each sector absorbed the MSNP goals uniquely into their own plans and activities. Finding the right balance between the sector‐specific demands and multisectoral coordination was cited as challenging by some. In addition, budgetary constraints, particularly for the non‐nutrition sectors, were also challenging to the MSNP.

Several respondents emphasized the importance of MSNP implementation, especially at the subnational level: ‘In the absence of local government, the Local Development Officer and the Village Development Committees are responsible for government efforts at local level’ (Government). District‐level Nutrition and Food Security Steering Committees were formed and led by the chair of the District Development Committee who coordinated implementation, monitored progress and managed programme bottlenecks. However, a few respondents also expressed concern about the number of committees at the local level and the long‐term sustainability of these structures. Concerns were also raised about local capacity in recognition of the importance of a district‐led approach given the diversity of contexts and health outcomes across the country: ‘[we] need to make a district specific MSNP to move away from the one size fits all … [we] need to document how different sectors spend the money’ (Donor).

There was agreement that the MSNP connected government ministries and established a new nutrition architecture in Nepal at all administrative levels while challenging traditional vertical programmes; although respondents highlighted that it was too early to determine the effects of MSNP, there was a perception that ‘many people are looking at Nepal because multisectoral approaches are the talk of the day everywhere, but it's hard to do it’ (NGO). *Key lessons* from the MSNP process included working strategies across sectors, importance of government engagement, leadership and coordination: ‘people underestimate what coordination requires and there is a lack of understanding in the difference between technical work and coordination’ (INGO). Several respondents also discussed the importance of reviewing MSNP to take stock of implementation and identify lessons that could be used to improve the plan.

### 3.2 ONA: Network linkages and dynamics

#### 3.2.1 Overall MSNP network

**Figure 2:**
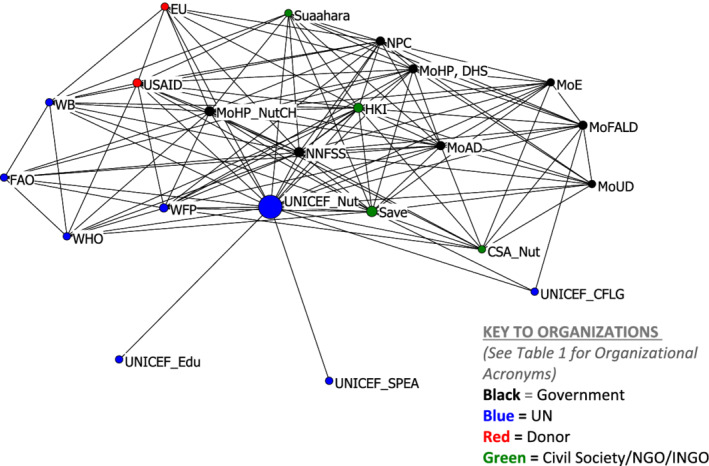
Confirmed Multisectoral Nutrition Plan whole network (nodes sized by betweenness centrality)

To explore the overall MSNP network connectivity, we assessed the confirmed network plot (Figure 2), along with density (extent that all potential connections are realized) and degree of centralization (potential power of direct relationships). These latter two measures of network closure are important features affecting the internal process of organizing the performance of policymaking networks (Burt, 2000).

Most of the 22 organizations in the network confirmed relationships with each other (330 out of 462 possible combination of ties) with only a few organizations not reciprocating their relationships. Due to a high density (53.3%), the centralization score was 51.4%, indicating that the MSNP network is not dependent on one or a few organizations to act as intermediaries in conveying the plans and activities of the network (Table 3).

**Table 3:** Density and centralization of MSNP network

	Density (%)	Centralization (%)
Whole MSNP network	53.3	51.4
Policy dialogue and development	42.0	63.8
Strategic planning	38.1	57.6
Scale‐up implementation	31.2	49.5

Figure 2 shows UNICEF's Nutrition Section as the main broker for fostering linkages between organizations that do not have a direct relationship with each other, represented as the largest ‘node’ or highest betweenness centrality. UNICEF Nutrition also had the highest degree centrality (21 connections) along with the 18 connections for Nepal's National Nutrition and Food Security Secretariat (NNFSS), the government body serving as a secretariat to facilitate the implementation of the MSNP on behalf of the NPC (Table 4). Organizations also clustered together, such as government bodies on the right side, the UN organizations on the bottom left side of the plot and the donors and NGOs centred towards the middle of the plot (with a few exceptions).

**Table 4:** Degree centrality and betweenness centrality measures of MSNP network

Organization	Degree centrality				Betweenness centrality			
	Whole MSNP network	Policy dialogue	Strategic planning	Scale‐up implementation	Whole MSNP network	Policy dialogue	Strategic planning	Scale‐up implementation
Civil society								
CSA_Nut	10	9	4	3	0.39	0.78	0.08	‐
HKI	16	9	10	5	2.83	1.05	1.63	0.88
Save	15	13	8	6	4.12	3.89	0.48	1.25
Suaahara	11	9	10	7	0.45	0.67	1.61	0.68
Donor								
EU	8	5	3	3	0.09	0.05	0.10	‐
USAID	13	10	11	9	0.90	1.18	1.44	2.44
Government								
MoAD	15	10	11	6	1.83	0.9	2.08	0.67
MoE	10	10	9	9	0.29	0.93	0.85	4.16
MoFALD	11	8	9	7	1.88	1.81	2.78	1.76
MoHP, DHS	14	9	10	10	2.02	0.65	1.11	2.13
MoHP_NutCH	16	13	13	10	2.47	2.89	5.65	11.08
MoUD	9	7	6	6	0.23	0.52	0.07	0.68
NNFSS	18	15	15	15	3.90	5.94	7.00	16.62
NPC	14	14	12	14	1.64	4.79	1.77	9.05
UN								
FAO	8	5	5	2	0.29	0.08	0.33	‐
UNICEF_CFLG	3	2	2	1	0.05	‐	‐	‐
UNICEF_Edu	1	1	‐	1	‐	‐	‐	‐
UNICEF_Nut	21	21	19	16	26.13	35.38	29.22	33.70
UNICEF_SPEA	1	1	1	1	‐	‐	‐	‐
WB	11	6	5	5	0.81	0.40	0.47	‐
WFP	12	9	10	7	0.89	1.09	2.29	3.01
WHO	9	8	3	1	0.24	0.79	0.10	‐

The Nutrition Section of the Child Health Division (NutCH) of the Department of Health Services (DHS), MoHP, is well integrated with all organization types, including UN agencies, donors and international NGOs, and is positioned closely to the NNFSS. The Ministry of Agriculture Development (MoAD) has the highest degree centrality (15 connections) of the non‐health government sectors and is a conduit for connecting non‐health sector ministries such as the Ministries of Education (MoE), Federal Affairs and Local Development (MoFALD) and Urban Development (MoUD), across many different types of organizational categories (including Civil Society, UN agencies other than UNICEF Nutrition and donors). MoFALD has fewer direct relationships than MoAD but plays a similar role in providing a communication bridge to organizations not directly connected with each other (as they both have a betweenness centrality score of 1.8) (Table 4). Save the Children, Helen Keller International and the Suaahara Project are more embedded with the Government of Nepal side of the network while continuing to maintain ties with others.

#### 3.2.2 Working relationships

**Figure 3:**
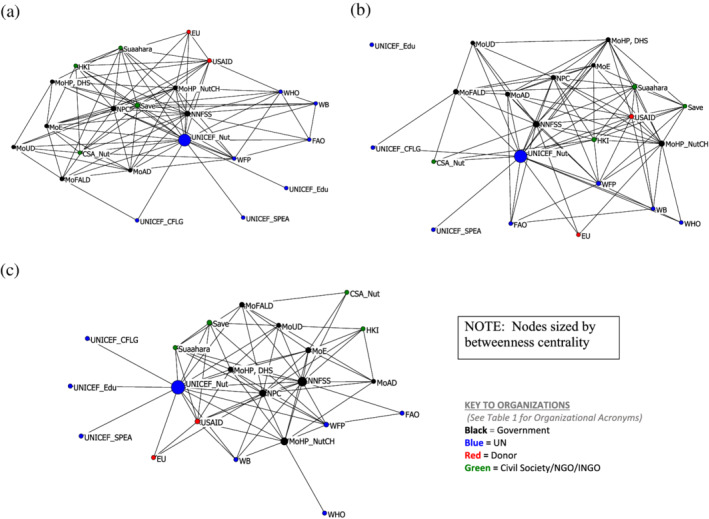
Multisectoral Nutrition Plan working relationships: (a) Policy dialogue and development, (b) Strategic planning, and (c) Scale‐up implementation

The MSNP planning process started with advocacy to make the case for multisectoral collaboration to address child stunting with a package of nutrition‐specific and nutrition‐sensitive interventions. This led to a policy dialogue to develop consensus on the major goals and objectives followed by strategic planning activities to develop the MSNP and the implementation and scale‐up of multisectoral activities (Figure 3a,c). The density of relationships and centralization are the highest for policy dialogue, followed by strategic planning and implementation (Table 3). The central broker and highest number of direct connections in all three types of linkages is the UNICEF Nutrition Section (Table 4). This illustrates UNICEF's supportive role to the government through the creation of bridges between organizations that enabled transmission of ideas and work to foster a unified understanding and potential for joint action.

Although the structure of the networks appears similar, there are distinct ways in which the organizations were positioned to fulfil different roles and responsibilities. For policy dialogue and development of the MSNP, the NPC and NNFSS both played a central role (Figure 3a). The donors, USAID and EU, were on the periphery whereas the NGOs and Suaahara Project that donors funded were located within the inner circle of government partners. This suggests that the donors did not have direct working relationships with NGOs on MSNP policy dialogue and development beyond the important role of providing resources.

The NNFSS continued to have a high number of direct ties with others while also serving as a central command post in maintaining channels of communication with organizations that had fewer direct relationships for strategic planning activities (Figure 3b and Table 4). The government ministries clustered together with the exception of the MoHP Nutrition Section. The MoHP Nutrition Section had direct relationships with multisectoral ministries (MoUD, MoE and MoAD) and the MSNP coordinating structure (NNFSS). The DHS was also connected with the donors, NGOs and the Suaahara Project.

For MSNP implementation and scale‐up (Figure 3c), there was lower organizational involvement in the network. The central government with NNFSS as the governance structure demonstrated an important brokerage role for the non‐health government ministries as well as some of the UN agencies (FAO, WFP and WHO) with a betweenness centrality score of 16.2. The MoHP_NutCH and the MoE (betweenness centrality scores of 11.1 and 4.2, respectively) also forged linkages with organizations not directly connected. UNICEF Nutrition worked with almost all organizations on implementation and scale‐up and facilitated the inclusion of other UNICEF programmes and NGOs in the network (Table 4).

#### 3.2.3 Relationship strength

**Figure 4:**
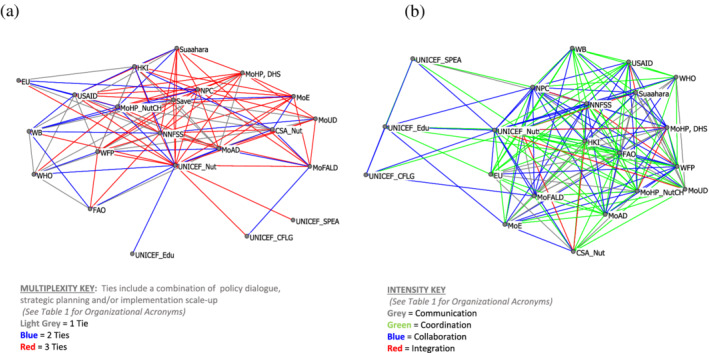
Multisectoral Nutrition Plan strength of organizational relationships: (a) Multiplexity and (b) relationship intensity

In examining the strength of relationships, the concept of multiplexity (participation across the three types of working relationships) and relationship intensity were measured. Figure 4a shows that the majority of relationships are for all three ties (red lines) signifying a very robust MSNP network that involved most organizations in working together. The level of intensity of relationships is presented in Figure 4b. The colour of the lines depicts the strength of the ties. The most basic relationship is communication (grey), and only a few organizations were at this first stage of relationship development. The majority of relationships were based on coordination (green) followed by collaboration (blue). Most of the collaboration occurred between NPC, NNFSS, different ministries and UNICEF Nutrition.

## 4 DISCUSSION

This study examined the structure and relationship dynamics of a multisectoral nutrition network in developing Nepal's first MSNP. We studied (1) three specific types of MSNP working relationships (policy dialogue, strategic planning and implementation and scale‐up), (2) the strength of multiorganizational collaboration and (3) the intensity of relationships. We find that the overall MSNP network of organizational actors was highly connected with specialized roles for the three types of working relationships that progressed through the stages of development and implementation of MSNP.

The NPC and the NNFSS that supported the NPC as the Secretariat for MSNP implementation were legitimate coordination and governance structures for policy dialogue, planning and implementation. Their central positions in the network indicate that the government played a leadership role in the stewardship of the MSNP. The NPC's location under the Prime Minister's office exerted influence for multisectoral collaboration with government as they approve the annual plans of each line ministry. The high degree of direct relationships, as depicted by the ONA relationship intensity measure, created a robust platform for coordination and collaboration. Nepal's densely linked network is an advantage as studies have demonstrated greater efficiency of information diffusion to all network members when compared with sparsely linked groups (Mays & Scutchfield, 2010).

We also uncovered challenges in the network structure and relationship patterns. The organizations in the network tended to cluster together by their own type such as the UN agencies and government ministries. This finding fits with the homophily principle of networks in which people and organizations with similar characteristics tend to stick together (Yuan & Gay, 2006). It is more difficult to achieve multisectoral collaboration when organizations of similar traits form cliques or clusters among themselves and are not building connections across different clusters.

Another major challenge was that ministries, with the exception of the MoHP, had variable direct connections among themselves or with donors, UN agencies and NGOs. MoE, MoFALD and MoUD relied on the MoAD to play an important bridging role in transmitting information about MSNP strategic planning activities whereas the MoE played a larger role in implementation and scale‐up of MSNP. UNICEF's Nutrition Section played a consistent role in linking in other UN agencies that did not have direct relationships into the network.

Prior research has recognized that the structure of relations among actors and the location of individual actors in the network have important behavioural and attitudinal consequences for the individual units and for the system as a whole (Knoke, 1994) and can close ‘structural holes’ or gaps in a network (Yuan & Gay, 2006). The structural holes theory posits that organizations (or people) that bridge the holes in direct linkages possess more social capital because they have access to a more diversified group of organizations that can lead to more opportunities and better performance (Burt, 2000; Varda, 2011). The highest bridging roles or ‘boundary spanning actors’ were NNFSS and UNICEF Nutrition Section. Social capital is enhanced by sharing information, facilitating common understanding and generating trust and commitment (Pelletier et al., 2018). However, brokers who become the conduits of knowledge exchange must balance their positions and not become overwhelmed by their brokering role (Long, Cunningham, & Braithwaite, 2013).

Collective action is often considered a success criterion in networks (Kickert, Klijn, & Koppenjan, 1997). Whereas the MSNP network of organizations had strong connectivity in the policy dialogue and the strategy development of the MSNP, the implementation and scale‐up displayed the weakest linkages. This finding was aligned with an earlier study conducted in Nepal that cited low nutrition capacity at district and community levels as a challenge to scaling up the MSNP interventions (Shrimpton et al., 2014). Globally, studies have also identified common difficulties such as the lack of incentives for non‐health sectors to coordinate for nutrition, poor or non‐existent mechanisms for accountability and limited functional vertical and horizontal coordination at district and community levels (Beyero, Hodge, & Lewis, 2014; Warren & Frongillo, 2017).

The Government of Nepal is currently implementing the second phase (2018–2022) of the MSNP, which continues to involve multiple sectors and stakeholders (Suresh et al., 2019). There are indications that a lack of a local infrastructure and knowledge sharing for cross‐institutional inputs is a barrier in implementation of the MSNP II (Gaihre et al., 2019). However, the replacement of a unitary government with a federal system of government in late 2017 is altering the implementation landscape (Bhattarai, 2019). The new structure offers an opportunity to change the centralized nature of programmes and financing of multisectoral nutrition services at the local level. This process requires attention to local multisectoral collaboration structures and process dynamics that power local accountability systems and community engagement. Significant investments in training for front‐line workers as well as subnational sectoral programme leaders and logistical support are necessary to ensure consistent programme implementation as well as autonomy of lower level actors to adjust operating procedures to address local needs (Beyero et al., 2014; Warren & Frongillo, 2017).

Our study has several limitations. Using qualitative methods and ONA cannot ascribe causality and be generalized to other settings. The use of ONA to assess multisectoral collaboration is a new area of work with no established standards of what constitutes a strong or a weak network. Although there is growing evidence that concepts such as density may influence the performance of partnership and networks, their exact mechanisms are not well known (Mays & Scutchfield, 2010). Some network measures such as density are affected by network size and may not be directly comparable across networks with substantially different structures.

Our selection of organizations and respondents was based on generating participants from a few key organizations and may have potentially excluded the less connected groups. However, we asked organizational respondents to include other organizations that they worked with that were not on a generated list of respondents. This process yielded very few others. As the study did not involve organizational respondents at the subnational level, this may have affected the perceptions about the subnational implementation networks in Nepal. Organizational interviews required the selection of an individual that was deemed knowledgeable about their organization's relationships with others. It is possible that some respondents did not have full knowledge of these organizational interactions. Lastly, the study lead/interviewer was employed by UNICEF, and controls were put into place to reduce potential bias. This included an informed consent process, no UNICEF staff were present during the interviews and the data were not accessible to UNICEF.

## 5 CONCLUSIONS

Multisectoral collaboration is a central strategy for the achievement of the SDGs and of the global SUN movement but has remained an elusive concept for many countries. Despite the development of multisectoral nutrition strategies and plans across the globe, there is a shortage of empirical research on multisectoral collaboration to improve nutrition outcomes. We find that the development and implementation of the first MNSP in Nepal was characterized by a high degree of multisectoral and multi‐stakeholder collaboration. Our study contributes to the small but growing literature that illustrates how ONA can be applied to measure, assess and use network results to understand the processes and dynamics to strengthen multisectoral planning and implementation.

## CONFLICTS OF INTEREST

The authors have no conflict of interest.

## CONTRIBUTIONS

JR designed the research study and conducted all interviews. AB analysed qualitative data, and CM performed ONA and developed visual plots in accordance with an analytical plan developed by JR, CM and AB. JR, HT, AB and CM wrote sections of the paper. HT provided guidance on the framing of this paper. All authors edited the paper.

## Data Availability

The data that support the findings of this study are available from the corresponding author upon reasonable request.
